# Titin As the Culprit Behind Dilated Cardiomyopathy: A Case Series of Three Cases and a Comprehensive Literature Review

**DOI:** 10.7759/cureus.67489

**Published:** 2024-08-22

**Authors:** Binay K Panjiyar, Nikita Changlani, Saroj K Jha, Sanam W Khan, Omar Khan

**Affiliations:** 1 Research, Ventolini's Lab, Texas Tech University Health Sciences Center, Odessa, USA; 2 Global Clinical Scholars Research Training, Harvard Medical School, Boston, USA; 3 Internal Medicine, California Institute of Behavioral Neurosciences and Psychology, Fairfield, USA; 4 Internal Medicine, Paul L. Foster School of Medicine, El Paso, USA; 5 Internal Medicine, Tribhuvan University Teaching Hospital, Kathmandu, NPL; 6 Internal Medicine, CMH Lahore Medical College, Lahore, PAK; 7 Internal Medicine, CMH Multan Institute of Medical Sciences, Multan, PAK

**Keywords:** heart failure, titin, case-series, dilated cardiomyopthy, titin gene

## Abstract

Nonischemic dilated cardiomyopathy (DCM) is a complex cardiovascular condition often characterized by genetic pathogenesis. Comprehensive genetic testing has become a crucial aspect of DCM diagnosis and management, offering insights into prognosis and the identification of at-risk individuals. We delve into distinct genetic pathways associated with DCM and their pathogenetic mechanisms, emphasizing the evolving significance of genetic markers, particularly in cases where arrhythmia risk is heightened. The historical reliance on cardiac morphology to subtype cardiomyopathies is being complemented by the identification of genetic variants, further refining DCM subtypes and aiding in clinical management. The first case is a 51-year-old male who presented with symptoms of heart failure and non-sustained ventricular tachycardia. The second case is a 65-year-old female who presented with chest pain, shortness of breath, and tachycardia-mediated cardiomyopathy. The third case was a 48-year-old male who had a history of heart failure and non-ischemic cardiomyopathy. Despite immediate and extensive resuscitative measures, the patients' protracted response to the treatment raised questions about the potential underlying genetic factors contributing to their clinical presentation. A genome study was done in all these reported cases, which showed a genetic mutation in the titin gene. These cases underscore the importance of genetic evaluation in unraveling the complexity of cardiomyopathies, ultimately enhancing our ability to manage and treat such challenging cases. This case series, with a comprehensive literature review, explores the mechanisms governing titin-based forces in healthy and diseased conditions. It highlights the influence of isoform diversity and post-translational modifications on myocardial stiffness and contractility.

## Introduction

Dilated cardiomyopathy (DCM), a condition characterized by dilatation of the left ventricle and impaired systolic function, has multifaceted etiologies. Among these, titin-truncating variants (TTNtvs) have gained prominence [1]. These variants are increasingly recognized as a common genetic cause of DCM, adding to the complexity of understanding the disease. This relationship between TTN and DCM has stirred intense debate regarding the precise mechanisms by which TTNtvs contribute to cardiomyopathy, whether through haploinsufficiency, dominant negative effects, or other mechanisms [1,2].

The discovery of TTNtvs as a common genetic cause of DCM has sparked intense research and debate within the scientific community. While TTNtvs play a significant role in DCM, several controversies and challenges have emerged, which are highlighted in the seminal studies by Roberts et al. and Schafer et al. [3,4].

This review delves into the multifaceted world of TTN and its pivotal role in DCM pathogenesis. It explores the evolving understanding of TTN's function, genetic variations, and clinical implications in DCM, shedding light on the complex interplay between this giant molecule and cardiac health.

## Case presentation

Case 1

A 51-year-old Hispanic male patient with heart failure, New York Heart Association (NYHA) classes II-III, presented to our center for a three-month follow-up with significant improvement of symptoms after guideline-directed medical therapy. The patient denied any history of chest pain, shortness of breath at rest or on exertion, syncope, palpitations, loss of consciousness, or claudication.

He had been previously diagnosed with chronic systolic heart failure. However, the ejection fraction improved after a single-chamber automated implantable cardioverter defibrillator (AICD) implant. There was a history of non-sustained ventricular tachycardia, premature ventricular contractions, and ventricular couplets. Other risk factors for coronary artery disease, such as obesity, hypertension, type II diabetes mellitus, and dyslipidemia, were also present. He had a history of quadruple bypass done five years back. He was taking valsartan 160 mg twice daily, amiodarone 200 mg once daily, amlodipine 5 mg once daily, carvedilol 25 mg twice daily, furosemide 20 mg once daily, hydralazine 25 mg twice daily, rosuvastatin 20 mg once daily, spironolactone 25 mg once daily, aspirin 325 mg once daily, empagliflozin 25 mg once daily, omega-3 polyunsaturated fatty acids 500 mg twice daily, and cholecalciferol 2000 IU once daily. He had a family history of myocardial infarction in his mother, congestive heart failure in two brothers and a sister at 57, 54, and 57 years, respectively, diabetes mellitus in his sister, murmur in his 15-year-old daughter, and autism in his son. He was a non-smoker, non-drinker, and had no known allergies.

**Figure 1:**
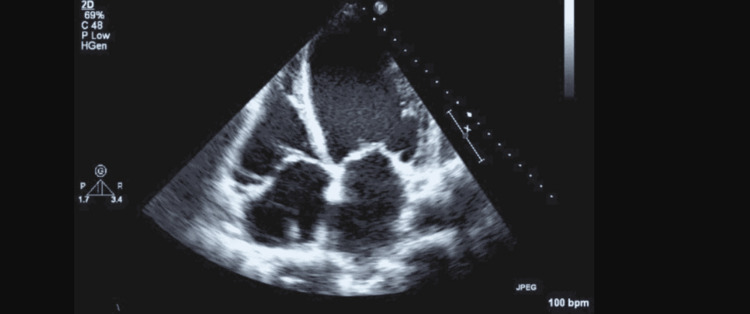
Case 1: Apical four-chamber echocardiographic view demonstrating dilated cardiomyopathy with left ventricular dilation and an ejection fraction of 40-45%.

His examination showed a pulse rate of 65 beats/minute, blood pressure of 112/75 mmHg, and BMI of 35.2. Heart and lung examinations were within normal limits. Echo showed an ejection fraction of 40-45% (Figure 1), mild mitral regurgitation, and aortic stenosis. A cardiometabolic genome test was ordered to evaluate the genetic basis for cardiac arrhythmias. The result showed a heterozygous c.68885_68888dupATAC (p. lle22964TyrfsTer8) likely pathogenic variant in the TTN (NM_001267550.1) gene. This was indicative of titin cardiomyopathy due to the genetic inheritance of a mutation in the TTN gene. In addition, this patient also had ischemic cardiomyopathy due to acquired coronary artery disease.

He was advised to continue the anti-diabetic diet, Dietary Approaches to Stop Hypertension (DASH) diet, and as-tolerated exercise. Medications were adjusted to sacubitril-valsartan in place of valsartan. Counseling was done regarding genetic testing of family members.

Case 2

A 65-year-old Hispanic female patient presented with right-sided chest pain, not precipitated by exertion, every day to every other day, not relieved by paracetamol. She also had shortness of breath on and off and dyspnea on exertion. The patient denied any history of syncope, orthopnea, palpitations, loss of consciousness, dizziness, or claudication.

The patient had a history of tachycardia-mediated cardiomyopathy (due to atrial fibrillation with fast ventricular rate) and pulmonary hypertension. In addition, she had a history of unprovoked acute deep vein thrombosis complicated with acute pulmonary embolism, which was treated with catheter-directed thrombolysis. Other risk factors for cardiac disease, such as hypertension, type II diabetes mellitus, and obesity, were also present. She was taking carvedilol 12.5 mg twice daily, losartan 50 mg once daily, omega-3 polyunsaturated fatty acids (fish oil 1000 mg) once daily, and rivaroxaban 10 mg once daily. She had a family history of myocardial infarction in her brother, who died at 60 years of age. She was a non-drinker, an ex-smoker who quit more than a decade ago, and had no known allergies.

**Figure 2:**
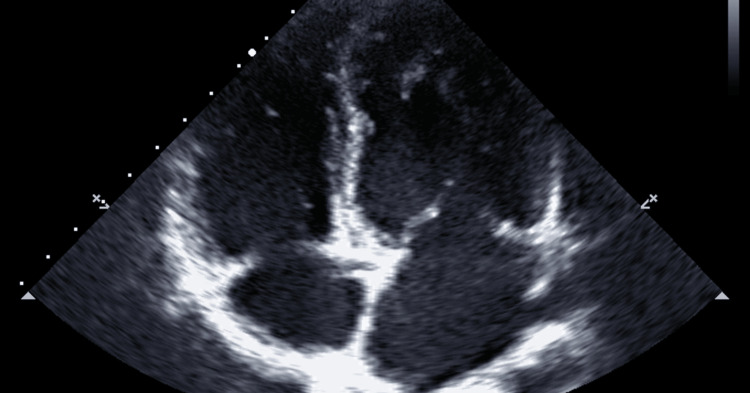
Case 2: Echocardiographic view showing dilated cardiomyopathy with marked left ventricular enlargement and a reduced ejection fraction of 35-40%.

Her examination showed a pulse rate of 76 beats/minute, blood pressure of 109/66 mmHg, and BMI of 39.2. Heart and lung examinations were within normal limits. The echo showed an ejection fraction of 35-40% (Figure 2). Holter monitor showed normal sinus rhythm and two episodes of paroxysmal atrial fibrillation with a rapid ventricular rate; the longest run was 10 seconds at 140 beats/minute. ECG performed on the day of examination showed normal sinus rhythm. The Treadmill Exercise Stress Test was negative for ischemia. Nuclear Lexiscan Stress Test was planned to evaluate for coronary artery disease.

A genome study was requested to evaluate for genetic mutations that may predispose patients to cardiovascular risks. The result showed a heterozygous c.75138_75141del (p. Lys25046Asnfs*8) likely pathogenic variant in the TTN (NM_001267550.1) gene. This was indicative of titin cardiomyopathy due to the genetic inheritance of a mutation in the TTN gene.

She was advised to continue the anti-diabetic diet and as-tolerated exercise. Medications were adjusted to rosuvastatin in place of atorvastatin, apart from guideline-directed medical therapy. She was made aware of the risk of passing the disease to future generations, and therefore, there was a need for genetic testing of family members. A follow-up was scheduled to review the report of the stress test.

Case 3

A 48-year-old Hispanic male patient with a history of heart failure (baseline ejection fraction of 15%) presented to our center for a follow-up. The patient denied any history of chest pain, shortness of breath at rest or on exertion, syncope, orthopnea, paroxysmal nocturnal dyspnea, palpitations, loss of consciousness, dizziness, or claudication.

The patient had a history of non-ischemic cardiomyopathy, which was treated in line with alcohol-induced cardiomyopathy, and the ejection fraction improved to 30%. He also had ventricular tachycardia, which was controlled on amiodarone and confirmed by AICD interrogations. Other risk factors for cardiac disease, such as hypertension, dyslipidemia with low HDL, obesity, and heavy alcohol abuse before quitting four years ago, were also present. He is currently on guideline-directed medical therapy, including amiodarone, aspirin, carvedilol, rosuvastatin, and spironolactone. He had a family history of hypertension and stroke in his father. He was a non-smoker, consumed beer one to two times per week, and had no known allergies.

**Figure 3:**
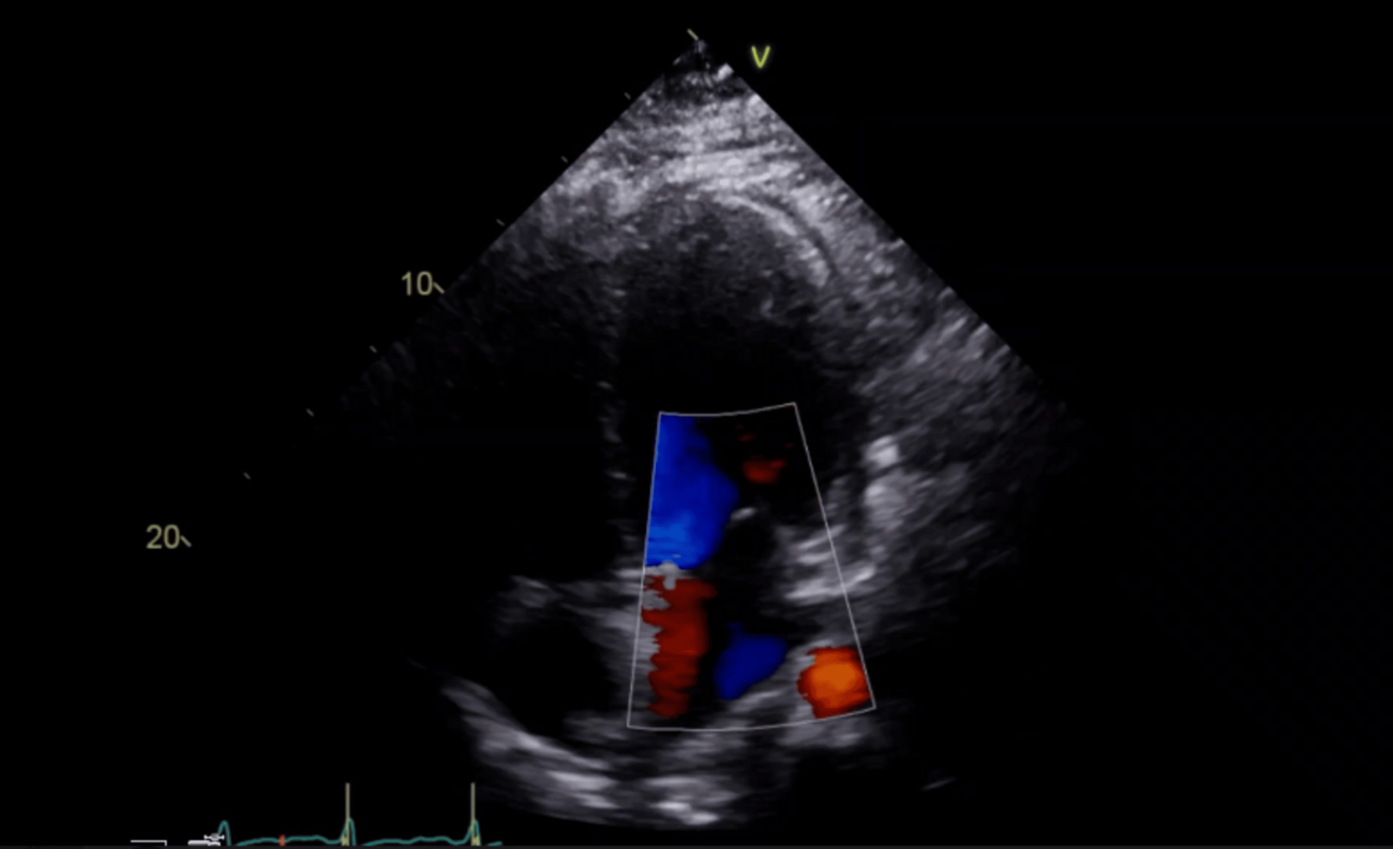
Case 3: Apical four-chamber echocardiographic view illustrating severe dilated cardiomyopathy with significant left ventricular enlargement and a critically reduced ejection fraction of 30%.

His examination showed a pulse rate of 66 beats/minute, blood pressure of 116/77 mmHg, and BMI of 31.1. Heart and lung examinations were within normal limits. ECG on the day of examination showed normal sinus rhythm. The echo showed an ejection fraction of 30% (Figure 3). AICD check showed two events of possible supraventricular tachycardia at 180 beats/minute, lasting 15 seconds. Cardiac catheterization showed normal coronary arteries.

A genome study was requested to evaluate for genetic mutations that may predispose patients to cardiomyopathy. The result showed a heterozygous c.67495C>T (p. Arg22499Ter) pathogenic variant in the TTN (NM_001267550.1) gene. This was indicative of titin cardiomyopathy due to the genetic inheritance of a mutation in the TTN gene.

He was advised to continue the low-carbohydrate diet, DASH diet, and as-tolerated exercise to lose weight. Sacubitril-valsartan was added. Genetic counseling was done on the patient alongside the family members. A follow-up EKG after four months was scheduled.

## Discussion

Titin, encoded by the gene TTN, is the largest protein in the human body, which is crucial for maintaining the structural and functional integrity of the sarcomere, spanning from the Z-disk to the M-band [1]. The complex cytoarchitecture of striated muscle, with its precise organization and coordination, is essential for the impressive contractile properties of these tissues. At the core of this complexity is titin, a massive protein extending across the sarcomere from the Z-disk to the M-band, playing a key role in maintaining muscle integrity and function. While the structural role of TTN is well-known, its involvement in cardiac pathophysiology, particularly in DCM, has become a major focus in cardiovascular research. TTN is the largest protein expressed in the heart, comprising approximately 35,000 amino acids. Its size and extensive alternative splicing, particularly in the I-band region, make it a challenging subject to study. TTN is essential for sarcomere contraction and signaling, functioning as a molecular spring and providing passive force. The specific isoforms of TTN also affect ventricular compliance, adding another layer of complexity.

DCM is a complex cardiovascular disorder characterized by the enlargement of the left ventricle and impaired systolic function, leading to heart failure and arrhythmias. Although the etiology of DCM is varied, one genetic factor that has received significant attention is truncations of the TTN, as shown in the pivotal study by Herman et al. [2]. They found that truncating mutations of the TTN gene were associated with DCM. These mutations result in the premature termination of protein synthesis, leading to a truncated titin protein or its absence. The study revealed that individuals with TTNtv have a significantly increased risk of DCM. A key finding of this study was the high prevalence of TTNtv in patients with idiopathic or familial DCM. About 25% of familial idiopathic DCM cases and 18% of sporadic cases were found to carry TTNtv [2]. These statistics highlight the significance of TTNtv as a genetic contributor to DCM, especially in cases with a familial predisposition. Notably, TTNtv was identified as an independent risk factor for DCM, with a fourfold increase in the odds of developing the disease. This underscores the critical role of TTN in maintaining normal cardiac structure and function. The study also noted that TTNtv was the most common genetic cause of DCM identified to date.

The mechanisms by which TTNtv leads to DCM have been a subject of ongoing research and debate. One hypothesis is the haploinsufficiency model, where the loss of one functional TTN allele results in insufficient titin protein production, compromising sarcomere function and contributing to DCM. Another hypothesis is the "poison peptide" model, where the truncated titin protein itself, encoded by the TTNtv-bearing allele, may interfere with sarcomere function and integrity. Understanding the functional consequences of TTNtv has been crucial. Herman et al. noted that TTNtv-bearing individuals exhibited variable expressivity of DCM, suggesting that factors beyond genetics contribute to the disease's development and progression. It was also observed that TTNtv was more frequently associated with a milder form of DCM, indicating that additional genetic modifiers or environmental influences may modulate the severity of the disease.

One of the primary controversies regarding TTNtvs in DCM involves their penetrance and expressivity. Roberts et al. conducted an extensive analysis, integrating allelic, transcriptional, and phenotypic data, to uncover the complex effects of TTNtvs. They found that TTNtvs are not solely deterministic of DCM. Instead, they observed a wide range of clinical outcomes among TTNtv carriers, from asymptomatic individuals to those with severe DCM. This finding challenges the notion that TTNtvs directly result in a uniform DCM phenotype. The interplay of genetic and environmental factors, along with additional genetic modifiers, likely contributes to the variable expressivity of TTNtvs in DCM. This complexity has led to ongoing discussions about predicting the clinical trajectory of TTNtv carriers and the implications for genetic counseling and clinical management [3]. Another contentious issue involves the distinction between TTNtvs as pathogenic or benign variations. Schafer et al. [4] explored this matter by examining TTNtvs in both disease cohorts and the general population. They discovered that TTNtvs were not exclusive to DCM patients but were also present in individuals without cardiac disease. This observation challenges the traditional dichotomy of pathogenic and non-pathogenic variants, suggesting that TTNtvs may not always result in clinical manifestations of DCM. Therefore, determining the clinical significance of TTNtvs becomes complex, raising questions about the necessity and appropriateness of genetic testing in asymptomatic individuals carrying TTNtvs. Labeling TTNtvs as pathogenic could lead to unnecessary psychological distress and overtreatment.

There is ongoing debate regarding the precise mechanisms by which TTNtvs contribute to DCM. While haploinsufficiency, where the loss of one functional TTN allele leads to insufficient titin protein production, is a leading hypothesis, the exact pathophysiology remains elusive. The "poison peptide" hypothesis, suggesting that the truncated titin protein itself interferes with sarcomere function, continues to be investigated. Understanding these mechanisms is essential for developing targeted therapies. Research efforts are underway to decipher the complex interactions between TTNtvs and other genetic and environmental factors that ultimately lead to DCM.

Genetic testing has become essential for clinicians in assessing and managing DCM patients. This process typically begins with a thorough evaluation of a patient's family history and clinical symptoms, forming the basis for genetic testing [5]. Genetic testing for DCM involves analyzing a panel of genes linked to this cardiac condition, with TTN being particularly significant due to the high prevalence of TTNtvs in DCM patients. The advent of next-generation sequencing (NGS) technologies, including whole-exome sequencing (WES) and whole-genome sequencing (WGS), has revolutionized the detection of genetic variants like TTNtvs. These advanced techniques allow for the simultaneous analysis of numerous genes, making the identification of potentially pathogenic variants, including TTNtvs, highly efficient. Once data are generated, it undergo careful interpretation and reporting. Laboratories examine the genetic information to identify potential pathogenic variants, such as TTNtvs, which are then reported to the attending clinician. Genetic counseling helps individuals and their families understand the implications of TTNtvs, providing insights into inheritance patterns, clinical significance, and the potential impact of these variants on disease risk. This step is essential in ensuring that individuals are well-informed about their genetic makeup and can make educated decisions regarding their health, family planning, and lifestyle choices. For clinicians and genetic counselors, identifying TTNtvs in DCM patients or asymptomatic carriers within affected families has several critical clinical implications. First, it enables risk assessment, helping determine the need for closer monitoring of heart failure symptoms, lifestyle modifications, and potential therapeutic interventions. Second, it underscores the importance of genetic counseling, like a collection of detailed family history, evaluation of the risk of occurrence in family members, and education of the patient to promote informed choices and appropriate interventions. This equips carriers with the knowledge to navigate the potential consequences of TTNtvs.

Moreover, it encourages a more personalized approach to treatment. While specific therapies targeting TTNtvs are still under investigation, carriers may benefit from traditional heart failure treatments and lifestyle adjustments. This personalized approach can significantly improve the quality of life for affected individuals.

The detection of TTNtvs in DCM through genetic testing is crucial for understanding the genetic foundations of this condition. It aids in early diagnosis and risk stratification within families, identifying asymptomatic carriers who may require monitoring and preventive measures. Beyond its diagnostic value, genetic testing for TTNtvs paves the way for precision medicine. As genomics advances, understanding genetic variants like TTNtvs is vital for their potential application in tailored treatments, offering hope for more effective DCM management in the future.

A key mechanistic insight into TTNtvs is their adverse impact on sarcomere assembly and stability, which results in abnormal muscle structure. This disruption manifests in characteristic features of DCM, such as ventricular dilation and reduced contractile function. The compromised structural integrity of TTN due to these variants impairs muscle contraction, contributing to inefficient blood pumping by the heart [6,7]. Additionally, TTNtvs provoke a cellular stress response in cardiomyocytes. This includes activation of the unfolded protein response and cellular signaling pathways as cells attempt to manage mutated and misfolded TTN protein. Persistent stress can lead to cell death and fibrosis. Fibrosis, a common histological finding in DCM patients with TTNtvs, correlates with heightened risks of adverse outcomes, including sudden cardiac death [8].

The long-term prognosis of DCM patients with TTNtvs is another clinically significant aspect. While therapeutic approaches have advanced, managing DCM with TTNtvs remains challenging. Some patients experience progressive heart failure and adverse outcomes, whereas others respond well to treatment and have a more favorable prognosis [9]. Captur et al. investigate the prediction of sarcomere mutations in individuals with subclinical hypertrophic cardiomyopathy (HCM) [10]. Sarcomere mutations, such as TTNtvs, are recognized as the cause of HCM, which is marked by the thickening of the heart muscle. This research underscores the possible genetic overlap between HCM and DCM, highlighting the variability of TTNtvs. It suggests that individuals with TTNtvs may display different clinical symptoms based on genetic modifiers and environmental influences.

TTNtvs in DCM are genetically heterogeneous, and their clinical manifestations can extend beyond DCM to conditions such as HCM [11]. Molecular mechanisms involve the mis-splicing of TTN exons, leading to dysfunctional protein products. The therapeutic strategy of antisense-mediated exon skipping holds promise as a potential treatment for TTN-based DCM by restoring the correct splicing pattern and, consequently, cardiac function. These insights illuminate the complex genetic and molecular landscape of TTNtvs in DCM and pave the way for innovative therapeutic approaches to this challenging condition.

Echocardiography is the primary imaging tool for cardiac assessment, crucial for evaluating cardiac structure and function. DCM patients typically show left ventricular dilation and reduced ejection fraction. As noted by Towbin et al., some DCM cases are associated with left ventricular non-compaction (LVNC) [12], detectable via echocardiography. Cardiac magnetic resonance imaging (MRI) offers enhanced tissue characterization, which is useful for distinguishing DCM from other cardiomyopathies. The genetic aspect of DCM, emphasized by Hershberger et al. [13], is increasingly important. Genetic testing can identify pathogenic mutations in genes like TTN, crucial for genetic counseling and family screening. For patients at high risk of life-threatening arrhythmias, implantable cardioverter-defibrillators (ICDs) are recommended [14]. ICDs detect and terminate ventricular arrhythmias, providing a critical safety net for DCM patients. Cardiac resynchronization therapy (CRT) may also be recommended for those with conduction abnormalities, optimizing cardiac function further.

## Conclusions

The clinical implications of these cases underscore the pivotal role of genetic findings in managing complex cardiovascular diseases. Genetic testing provided critical insights, guiding personalized treatment strategies and informing family counseling. These cases highlight the importance of early genetic evaluation, especially in patients with atypical presentations or a strong family history of cardiomyopathy. The lessons learned emphasize the need for integrating genetic testing into routine practice, ensuring more precise diagnoses, and improving patient outcomes through tailored care. This approach fosters a multidisciplinary, patient-centered strategy that enhances the management of inherited cardiovascular diseases.
